# Antihypertensive Medications and Risk of Heat‐Related Illness During Heatwaves: Self‐Controlled Case Series

**DOI:** 10.1002/pds.70447

**Published:** 2026-09-08

**Authors:** Mia Harley, Masao Iwagami, Chitose Kawamura, Elizabeth Williamson, Christopher T. Rentsch, Rohini Mathur, Ian J. Douglas, Dorothea Nitsch, Yuta Taniguchi, Yoko Hamasaki, Ai Suzuki, Naoaki Kuroda, Jun Komiyama, Angel Y. S. Wong, Nanako Tamiya

**Affiliations:** ^1^ Department of Non‐Communicable Disease Epidemiology, Faculty of Epidemiology and Population Health London School of Hygiene and Tropical Medicine London UK; ^2^ Department of Digital Health, Institute of Medicine University of Tsukuba Tsukuba Japan; ^3^ Health Services Research and Development Center University of Tsukuba Tsukuba Japan; ^4^ Digital Society Division, Center for Cyber Medicine Research University of Tsukuba Ibaraki Japan; ^5^ Department of Health Services Research, Institute of Medicine University of Tsukuba Tsukuba Japan; ^6^ Department of Internal Medicine Yale School of Medicine New Haven Connecticut USA; ^7^ Wolfson Institute of Population Health, Queen Mary University of London London UK; ^8^ Department of Public Mental Health Research National Institute of Mental Health Kodaira Tokyo Japan

**Keywords:** antihypertensives, heat‐related illness, heatwaves, hypertension, self‐controlled case series

## Abstract

**Purpose:**

To investigate whether antihypertensive medications modify the risk of heat‐related illness during heatwaves among adults with hypertension in Japan.

**Methods:**

We identified adults with hypertension in Tsukuba City administrative claims data between April 2014 and March 2019. Average daily temperature was derived from Japan Meteorological Agency data. We used a self‐controlled case series (SCCS) design to estimate incidence rate ratios (IRRs) for heat‐related illness during 5‐day pre‐heatwave, heatwave and 5‐day post‐heatwave periods compared to baseline within individuals. The main analysis was stratified by person‐time prescribed antihypertensives (angiotensin‐converting enzyme [ACE] inhibitors/angiotensin II receptor blockers [ARBs], calcium channel blockers [CCBs] or non‐loop diuretics) and person‐time not prescribed antihypertensives, with drug‐temperature interaction assessed. Subgroup analyses were conducted for each antihypertensive class. All models were adjusted for age and season.

**Results:**

We observed a greater increase in the rate of heat‐related illness during heatwaves versus baseline among person‐time prescribed antihypertensives (IRR: 1.64, 95% CI: 1.53–1.75) than among person‐time not prescribed antihypertensives (IRR: 1.42, 95% CI: 1.27–1.58), with evidence of drug‐temperature interaction (*p* < 0.01). When examining individual drug classes, ACE inhibitors/ARBs and CCBs were associated with an increased risk of heat‐related illness during heatwaves, whereas there was no evidence of an effect with diuretics.

**Conclusions:**

Our findings indicate that people taking antihypertensives are more vulnerable to heat‐related illnesses during heatwaves. More research is required to explore whether this is a causal effect of the medication or due to differences between those who are treated and untreated with antihypertensives that are related to the outcome.

## Key Points

- In a self‐controlled case series including 11 211 adults with hypertension in Japan, we compared heat‐related illness event rates during heatwaves versus baseline with and without antihypertensives.
- We observed a greater increase in heat‐related illness event rate with antihypertensives (IRR: 1.64, 95% CI: 1.53–1.75) than without antihypertensives (IRR: 1.42, 95% CI: 1.27–1.58).
- The finding was consistent in subgroup analyses looking at ACE inhibitors/ARBs and calcium channel blockers, but not for non‐loop diuretics.
- These findings demonstrate greater vulnerability to heat‐related illness during heatwaves among people taking antihypertensives.
- More research is required to explore whether this is a causal effect of the medication.

## Plain Language Summary

Climate change is increasing the frequency and intensity of heatwaves worldwide. Older adults with morbidities, such as hypertension, are particularly at risk of heat‐related illness. Antihypertensive medications, used to treat hypertension, can affect how the body regulates temperature and fluid balance, which may increase heat‐related illness risk. In this study we compared the rate of heat‐related illness events during heatwave periods to normal temperature periods within an individual. We then compared the increase in heat‐related illness during heatwaves during time using antihypertensives and time not using antihypertensives. We found that there was a greater increase in heat‐related illness rates during heatwaves when taking antihypertensives than when not taking antihypertensives. When we looked at individual antihypertensive medication classes, we found that this pattern was consistent for angiotensin‐converting enzyme (ACE) inhibitors/angiotensin II receptor blockers (ARBs) and calcium channel blockers but not for non‐loop diuretics. This finding suggests that people who are taking antihypertensives are particularly vulnerable to heat‐related illness during heatwaves. However, more research is required to assess whether the increased risk is caused by the medication.

## 1 Introduction

Climate change has led to an increase in the frequency, intensity and duration of extreme weather events worldwide [1]. There is a well‐established link between increasing ambient temperatures and heat‐related illnesses, such as heat exhaustion, volume depletion, fluid and electrolyte imbalances and heat stroke [2]. Elderly adults and people with chronic conditions are particularly vulnerable to heat‐related illnesses [3, 4].

The mechanism of action of certain antihypertensive medication classes may increase vulnerability to heat‐related illness [5]. Angiotensin‐converting enzyme (ACE) inhibitors block the enzyme that converts angiotensin I into angiotensin II, which binds to various receptors stimulating the constriction of blood vessels, release of aldosterone from adrenal glands, retention of sodium and secretion of potassium from the urine, the reabsorption of water by the kidneys, the sensation of thirst and a desire to consume salt [6]. Angiotensin II receptor blockers (ARBs) block angiotensin II from binding to angiotensin 1 receptors in the heart, blood vessels and kidneys that stimulate vasoconstriction and aldosterone secretion to increase blood pressure [7]. CCBs inhibit the influx of calcium ions into vascular smooth muscle and cardiac cells, causing vasodilation and lowering blood pressure [7]. Diuretics promote excretion of water and electrolytes from the urine by suppressing sodium reabsorption into the renal tubules. The resulting higher concentration of sodium outside the tubules reduces osmotic reabsorption of water into the tubules [7]. While there are plausible biological mechanisms by which medications may increase risk of heat‐related illness, the extent to which these medications contribute to heat‐related illness during heatwaves in different settings is unclear.

We use a self‐controlled case series (SCCS) design to evaluate whether antihypertensives modify the risk of heat‐related illness during heatwave periods in Japan. We also explored how individual antihypertensive classes modified the effect of heatwaves on heat‐related illness.

## 2 Methods

### 2.1 Data Sources

We used National Health Insurance (NHI) and late‐stage medical care system administrative claims data from Tsukuba City in the Ibaraki prefecture of Japan, which includes medical procedures, prescription and diagnostic data. The NHI is for unemployed or self‐employed people and retirees aged 74 or younger. The late‐stage medical system covers all individuals aged 75 or older [8, 9, 10]. Administrative claims data were available from 1 April 2014 to 31 March 2019, which defined the study period. The analyses used only de‐identified patient‐level data, and therefore individual informed consent was not required. Publicly available climate data from the Japan Meteorological Agency (JMA) were used to estimate the mean daily temperature in Tsukuba City during the study period [11]. JMA measurements are recorded using an observation instrument located in the urban area of Tsukuba City, where most residents live [12]. While Tsukuba City covers an area of approximately 284 km^2^, the main urban area covers around 27 km^2^, therefore, the temperature at the observation station is expected to provide a reasonable estimate of the temperature throughout the main urban area [13].

### 2.2 Study Population

The study population was adults aged 18 years and over, with a record of hypertension diagnosis, using International Classification of Diseases 10th Revision‐10 (ICD‐10) codes (Table S1). A SCCS design selects participants who experience the outcome and have follow‐up spanning both exposure conditions. Therefore, to be included in the study population, individuals had to have been in the database during at least one heatwave period and have at least one diagnosis of heat‐related illness after being diagnosed with hypertension. For each person in the study population, the observation period started on the latest of the date they turned 18 years old, date of earliest diagnostic code for hypertension and the start of the study period, and ended on the earliest of death, the end of contributing to the database and the end of the study period. Data on the demographic and clinical characteristics of the study population at the start of their observation period were ascertained from inpatient and outpatient diagnostic datasets and prescription records.

### 2.3 Heatwave Exposure

Heatwaves were defined as ≥ 2 consecutive days during which the mean daily temperature exceeded the 95th percentile of that particular year [14]. The 5 days before and after heatwaves, defined as ‘pre‐heatwave’ and ‘post‐heatwave’ periods, respectively, were analysed separately to account for fluctuations in temperature and potential residual effects of extreme temperature exposure on the outcome. Where exposure windows overlapped, heatwave exposure periods took precedence, followed by post‐heatwave periods, then pre‐heatwave periods. The remaining time was defined as non‐heatwave periods.

### 2.4 Drug Exposure

The drug exposures were ACE inhibitors/ARBs, CCBs and non‐loop diuretics, which are the recommended first‐line and most prescribed antihypertensives in Japan [15, 16]. Loop diuretics were excluded because these drugs have a short half‐life and patients may be advised to stop taking them during heatwaves [17]. Prescriptions of exposure medications were identified using WHO Anatomical Therapeutic Chemical (ATC) codes (Table S3). Drug exposure periods were derived using the prescription issue dates, quantities and intended duration, allowing up to a 30 day gap in prescription records during continuous exposure periods.

### 2.5 Outcome

The outcome was heat‐related illness, which included heatstroke and sunstroke, volume depletion, disorders of fluid, electrolyte and acid–base balance and exposure to excessive natural heat [2]. These outcomes were chosen because they are acute outcomes that are directly caused by heat exposure, are well captured in the data sources used, and align with the definition of heat‐related illness in previous research [18, 19]. Heat‐related illness events were identified from inpatient and outpatient diagnostic datasets using ICD‐10 codes (Table S1). In the main analysis, multiple event occurrences were allowed in the same person, with a minimum of 30 days required between each event to be considered a separate event. Where an event occurred within 30 days of a previous event within the same individual, the later event was not counted as a separate outcome; however, the follow‐up time was still included in the analysis.

### 2.6 Study Design

**FIGURE 1:**
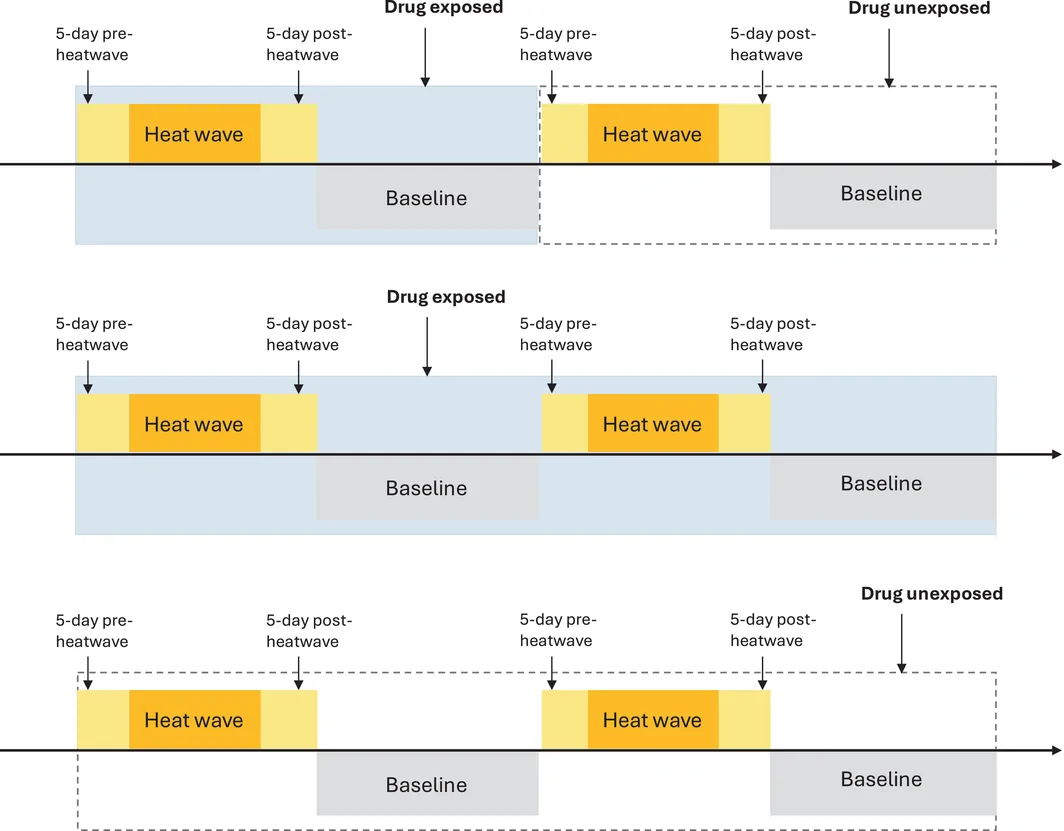
Diagram depicting the self‐controlled case series study design. The figure illustrates how follow up time from individuals was categorised in the self‐controlled case series analysis. The top panel depicts individual follow‐up that includes both drug‐exposed and drug‐unexposed periods. The middle panel depicts individual follow‐up entirely during drug‐exposed periods. The bottom panel depicts individual follow‐up entirely during drug‐unexposed periods. To be included in the analysis, individuals had to have experienced both a heatwave period and baseline temperature period.

The SCCS study design, as visually depicted in Figure 1, was used to compare the risk of heat‐related illness during pre‐heatwave, heatwave and post‐heatwave periods with non‐heatwave periods for person‐time with and without exposure to antihypertensives. This study design was chosen because it is suitable for examining the relationship between transient exposures and acute outcomes. SCCS is a case‐only study design where each individual acts as their own control; therefore, controls are not required, and time‐invariant confounding is intrinsically controlled for [20, 21].

### 2.7 Statistical Methods

Incidence rate ratios (IRRs) for heat‐related illness were estimated for pre‐heatwave, heatwave and post‐heatwave periods compared to baseline using conditional Poisson regression. Drug exposure was added to the model as an interaction term, which was used to derive IRRs for heat‐related illness separately for drug exposed and drug unexposed person time. A likelihood ratio test assessed whether there was statistical evidence of interaction between heatwave exposure and drug exposure. The main analysis was adjusted for season (winter: 1 December to 28 February; spring: 1 March to 31 May; summer: 1 June to 31 August and autumn: 1 September to 30 November), and 5‐year age group during follow‐up intervals.

### 2.8 Subgroup Analyses

We conducted separate SCCS analyses examining the effect of individual drug classes on the rate of heat‐related illness during heatwaves. In these analyses, exposure was defined by use of the specific drug class of interest, while intervals in which individuals were not prescribed this drug class were classified as unexposed. Where people were prescribed multiple antihypertensives or on co‐formulations, these split into their constituent parts and treated as separate prescriptions. Concurrent use of other antihypertensive classes (including other components of co‐formulations) during follow‐up intervals was adjusted for as a time‐varying covariate, along with season and 5‐year age group.

### 2.9 Sensitivity Analyses

We conducted several sensitivity analyses by repeating the main analysis looking at the interaction between antihypertensives and heatwave periods on the rate of heat‐related illness making the following adjustments one at a time. First, we excluded November to March from the observation period, when the temperature is lower (Table S5) therefore, heat‐related illness events are less plausible. Second, we reclassified person‐days in hospital that occurred during a heatwave period as unexposed to heatwaves, under the assumption that people are unlikely to be exposed to extreme temperatures while in hospital. Finally, we conducted separate SCCS analyses comparing baseline and heatwave periods irrespective of drug exposure in the total study population and stratified by age and sex, to assess the overall effect of heatwaves on heat‐related illness risk in our study population and potential effect modification by age and sex.

## 3 Results

### 3.1 Study Population

A total of 11 211 adults were identified with a diagnosis of hypertension who subsequently experienced a heatwave and had at least one heat‐related illness diagnosis during the study period. The median duration of observation period was 4.8 years (IQR: 3.0–4.8). Although exposure groups were overlapping, there was a larger sample contributing CCB (*n* = 8727) and ACE inhibitor/ARB (*n* = 7005) exposure than the diuretic exposure (*n* = 2908). People prescribed diuretics were on average older with more comorbid conditions than those prescribed ACE inhibitors/ARBs and CCBs (Table 1).

**TABLE 1:** Demographic and clinical characteristics of people that contributed to each drug exposure group and in the total study population at the start of the observation period.

	ACEi/ARBs	CCBs	Diuretics	All classes	Total study population
Sample	7005	8727	2908	10 163	11 211
Sex					
Male	3328 (47.5)	3922 (44.9)	1322 (45.5)	4575 (45.0)	5063 (45.2)
Female	3677 (52.5)	4805 (55.1)	1586 (54.5)	5588 (55.0)	6148 (54.8)
Age group					
18–40	52 (0.7)	74 (0.8)	18 (0.6)	82 (0.8)	113 (1.0)
40–50	129 (1.8)	135 (1.5)	47 (1.6)	171 (1.7)	207 (1.8)
50–60	308 (4.4)	358 (4.1)	100 (3.4)	409 (4.0)	462 (4.1)
60–70	1462 (20.9)	1720 (19.7)	451 (15.5)	2015 (19.8)	2231 (19.9)
> 70	5054 (72.1)	6440 (73.8)	2292 (78.8)	7486 (73.7)	8198 (73.1)
Average consultation months per year					
< 3	1154 (16.5)	1432 (16.4)	414 (14.2)	1689 (16.6)	1940 (17.3)
3–6	5061 (72.2)	6272 (71.9)	2141 (73.6)	7308 (71.9)	7999 (71.3)
> 6	790 (11.3)	1023 (11.7)	353 (12.1)	1166 (11.5)	1272 (11.3)
Pre‐existing comorbidities					
Ischaemic heart disease	1923 (27.5)	2415 (27.7)	1011 (34.8)	2825 (27.8)	3042 (27.1)
Heart failure	1228 (17.5)	1448 (16.6)	915 (31.5)	1826 (18.0)	2036 (18.2)
Venous thromboembolism	95 (1.4)	130 (1.5)	59 (2.0)	155 (1.5)	178 (1.6)
Cerebrovascular disease	2253 (32.2)	2803 (32.1)	1015 (34.9)	3190 (31.4)	3486 (31.1)
Type 2 diabetes	2683 (38.3)	3188 (36.5)	1185 (40.7)	3690 (36.3)	4015 (35.8)
Chronic renal disease	580 (8.3)	682 (7.8)	242 (8.3)	769 (7.6)	813 (7.3)
Chronic obstructive pulmonary disease	676 (9.7)	865 (9.9)	349 (12.0)	1039 (10.2)	1175 (10.5)
Cancer	844 (12.0)	1129 (12.9)	399 (13.7)	1345 (13.2)	1530 (13.6)
Depression and anxiety	919 (13.1)	1208 (13.8)	376 (12.9)	1379 (13.6)	1552 (13.8)
Severe mental illness	231 (3.3)	331 (3.8)	109 (3.7)	407 (4.0)	485 (4.3)
Medications used in 90 days prior					
Beta blockers	864 (12.3)	982 (11.3)	492 (16.9)	1184 (11.7)	1282 (11.4)
Statins	1693 (24.2)	1967 (22.5)	693 (23.8)	2310 (22.7)	2482 (22.1)
Glucose‐lowering medications	986 (14.1)	1119 (12.8)	441 (15.2)	1316 (12.9)	1417 (12.6)
Oral anticoagulants	356 (5.1)	422 (4.8)	289 (9.9)	548 (5.4)	601 (5.4)
Antibiotics	844 (12.0)	1118 (12.8)	405 (13.9)	1355 (13.3)	1557 (13.9)
Proton pump inhibitors	1804 (25.8)	2229 (25.5)	901 (31.0)	2650 (26.1)	2895 (25.8)
Antidepressants	176 (2.5)	234 (2.7)	77 (2.6)	273 (2.7)	307 (2.7)
Antipsychotics	181 (2.6)	300 (3.4)	99 (3.4)	363 (3.6)	428 (3.8)

Abbreviations: ACEi, angiotensin‐converting enzyme inhibitor; ARB, angiotensin II Receptor Blocker; CCB, calcium channel blocker.

### 3.2 Temperature in Tsukuba City

Between April 2014 and March 2019, 18 heatwaves were identified in Tsukuba City using JMA climate data. There were 2–4 heatwaves each year and the median duration of heatwaves was 3 days (IQR: 2–6) (Table S4). The median of mean daily temperature during heatwaves was 28.6°C (IQR: 28.0–29.4); the maximum daily temperature recorded during heatwaves ranged from 35°C to 37.4°C (Table S6).

### 3.3 Antihypertensives and Heat‐Related Illness

**FIGURE 2:**
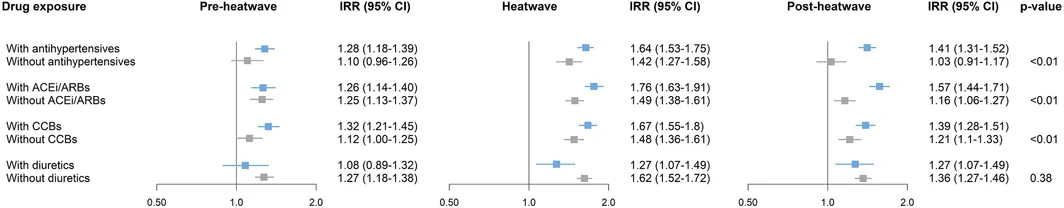
Forest plot of IRRs and 95% CIs for heat‐related illness during each heatwave period compared to baseline among person time prescribed antihypertensives and person time not prescribed antihypertensives. Each panel shows IRRs and 95% CIs for heat‐related illness during the 5‐day pre‐heatwave, heatwave and 5‐day post‐heatwave periods compared with baseline temperature periods. Estimates are shown separately for person‐time prescribed antihypertensives and person‐time not prescribed antihypertensives. The *p* value represents the statistical test for interaction between antihypertensive exposure and heatwave period. The overall antihypertensive analysis includes all three drug classes (ACEi/ARBs, CCB and diuretics). ACEi, angiotensin‐converting enzyme inhibitor; ARB, angiotensin II receptor blocker; CCB, calcium channel blocker; CI, confidence interval; IRR, incidence rate ratios.

There was evidence to suggest that antihypertensives modified the association between heatwaves and heat‐related illness, with higher IRRs observed with antihypertensives than without antihypertensives in heatwave periods compared to baseline (*p* < 0.01). During the pre‐heatwave period, the IRR for heat‐related illness compared to baseline was 1.28 (95% CI: 1.18–1.39) with antihypertensives, compared with 1.10 (95% CI: 0.96–1.26) without. When comparing heatwave periods to baseline, the IRRs were 1.64 (95% CI: 1.53–1.75) with antihypertensives and 1.42 (95% CI: 1.27–1.58) without. During the post‐heatwave period, the IRR for heat‐related illness compared to baseline was 1.41 (95% CI: 1.31–1.52) with antihypertensives compared to 1.03 (95% CI: 0.91–1.17) without (Figure 2).

### 3.4 Individual Drug Classes and Heat‐Related Illness

For ACEi/ARBs, there was evidence that medications modified the effect of heatwaves on rate of heat‐related illness, with higher IRRs observed with ACE inhibitors/ARBs than without during the heatwave and post‐heatwave periods (*p* < 0.01). During pre‐heatwave periods, there was a similar increase in rate of heat‐related illness compared to baseline among person‐time with (1.26, 95% CI: 1.14–1.40) and without (1.26, 95% CI: 1.14–1.38) ACE inhibitors/ARBs. When comparing heatwave periods to baseline, a larger IRR was observed among person‐time with ACE inhibitors/ARBs (1.77, 95% CI: 1.63–1.92) than without (1.50, 95% CI: 1.39–1.62). Similarly, when comparing post‐heatwave to baseline, the IRR was higher with ACEi/ARBs (1.57, 95% CI: 1.44–1.71) than without (1.17, 95% CI: 1.07–1.28) (Figure 2).

There was evidence that CCBs modified the effect of heatwaves on heat‐related illness, with consistently higher IRRs observed during person‐time with CCBs compared to without in heatwave periods compared to baseline (*p* < 0.01). When comparing pre‐heatwave periods to baseline, the IRR for person‐time with CCBs was 1.33 (95% CI: 1.21–1.46), whereas it was 1.13 (95% CI: 1.01–1.26) without. When comparing heatwave periods to baseline, the IRRs were 1.40 (95% CI: 1.29–1.51) with CCBs and 1.22 (95% CI: 1.11–1.34) without. For post‐heatwave periods compared to baseline, the IRRs were 1.40 (95% CI: 1.29–1.51) with CCBs and 1.22 (95% CI: 1.11–1.34) without (Figure 2).

During the pre‐heatwave period, the IRR for heat‐related illness compared with baseline was 1.08 (95% CI: 0.89–1.32) during person‐time with diuretics and 1.27 (95% CI: 1.18–1.38) without. During heatwaves, the IRRs were 1.59 (95% CI: 1.37–1.84) with diuretics and 1.62 (95% CI: 1.52–1.72) without. In the post‐heatwave period, the IRR was 1.27 (95% CI: 1.07–1.49) with diuretics and 1.36 (95% CI: 1.27–1.46) without. There was no statistical evidence of effect modification by diuretic use (*p* = 0.38) (Figure 2).

### 3.5 Sensitivity Analyses

When colder months (November–March) were excluded from the observation period, the findings were consistent with the main analysis findings (Table S7). When inpatient days were reclassified as ‘baseline’ temperature exposure, the findings remained consistent with those of the main analysis (Table S8). When we assessed the association between heatwave periods and heat‐related illness regardless of drug exposure, we found that the IRR of heat‐related illness increased during all heatwave periods in the total study population (heatwave IRR: 1.59, 95% CI: 1.50–1.69; pre‐heatwave IRR: 1.24, 95% CI: 1.16–1.34; post‐heatwave IRR: 1.33, 95% CI: 1.25–1.42, compared to baseline) and no evidence of interactions by sex (*p* = 0.89) or age (*p* = 0.98) (Table S9).

## 4 Discussion

### 4.1 Overall Findings

In this population‐based study, we found evidence of a 64% increase in the rate of heat‐related illness during heatwaves among person‐time prescribed antihypertensives compared to a 42% increase among person‐time not prescribed antihypertensives. When examining specific drug classes, prescription of ACE inhibitors/ARBs and CCBs was associated with an increased risk of heat‐related illness during heatwaves, whereas there was no evidence of a change in heat‐related illness rates with use of diuretics. The rate of heat‐related illness events with antihypertensives also increased during pre‐ and post‐heatwave periods more than without antihypertensives, suggesting that vulnerability may extend beyond formally defined heatwave periods.

### 4.2 Findings in Context

The observed increase in heat‐related illness during heatwaves and the magnitude of effect sizes in the present study for overall study population (IRR: 1.55) are in a similar range to those reported in previous studies [2], with IRRs of 1.45–1.64 reported for heat‐related illness during heatwaves in similar studies in Japan [18, 22].

Few studies have examined the role of antihypertensives as an effect modifier of heatwaves on heat‐related illnesses. A previous SCCS study using US Medicare data observed larger IRRs during heatwaves in drug exposed person‐time than in drug unexposed person‐time for both ACE inhibitors (1.42, 95% CI: 1.21–1.67 and 1.22, 95% CI: 1.07–1.38, respectively) and diuretics (1.52, 95% CI: 1.20–1.78 and 1.25, 95% CI: 1.11–1.41), but no clear evidence of interaction for either medication class (*p* = 0.20 and *p* = 0.33, respectively) [19]. Despite a similar study design and heatwave definition (≥ 2 consecutive days with a daily maximum above the 95th percentile regional temperature), differences in findings between this study and the present study may stem from contextual differences between the US and Japan such as climate, housing quality and drug utilisation patterns. A prescription‐event symmetry analysis reported increased rates of dehydration and heat‐related illness hospitalisations after initiating ACE inhibitors and diuretics, but did not assess interaction with heatwaves [23]. A French pharmacovigilance analysis during a heatwave found that ACE inhibitors and diuretics accounted for a large share of dehydration adverse drug reactions, but did not compare with non‐heatwave periods [24].

### 4.3 Individual Drug Classes

The higher rates of heat‐related illness observed during periods with ACE inhibitors/ARBs is consistent with their known physiological effects, which reduce thirst and desire for salt and reduce renal tubular sodium and water reabsorption, leading to dehydration [6]. The findings also align with previous research associating ACE inhibitors/ARBs with dehydration and heat‐related illness [23, 24]. The higher rates of heat‐related illness observed among person‐time with CCBs may be related to their cardiac muscle relaxant effects, which have been suggested to prevent increased cardiac output in response to thermal stress [25]. However, to our knowledge, the effects of these medications on heat‐related illnesses have not been previously evaluated. We observed no statistical evidence of an interaction for diuretics despite plausible mechanisms [7] and known dehydrating effects [26, 27]. Firstly, there may be unrecorded treatment breaks among diuretic users during summer months, given that treatment guidelines highlight dehydration risk with diuretics [17] although we excluded diuretics with a short half‐life. Secondly, the diuretic users were older and had more comorbidities than other antihypertensive medication users [28, 29], which may contribute to behavioural factors such as staying indoors Table 1.

### 4.4 Limitations

Firstly, although the SCCS design controls for time‐invariant confounding within individuals, there may be sources of time‐varying confounding that were not controlled for, for example changes in underlying health status may influence both antihypertensive use and susceptibility to heat‐related illness. Additionally, while SCCS controls for time‐invariant confounding when estimating within‐person incidence rate ratios, this does not extend to comparisons of incidence rate ratios between treated and untreated groups. Therefore, differences in effect estimates between treated and untreated groups may reflect underlying differences between these groups, rather than a causal effect of the medication. Nevertheless, our findings identify a high‐risk group during heatwaves, with important implications for patient safety. Secondly, we did not explore the different antihypertensive drug classes as mutually exclusive exposure categories within a single analysis, due to sample size constraints. Future research could explore this in a larger dataset. Thirdly, we identified people with hypertension using ICD‐10 codes only without using blood pressure measurements as this data was unavailable. Fourthly, drug exposure was defined using prescription records, which do not capture adherence or unrecorded treatment changes, resulting in potential drug exposure misclassification. Fifthly, temperature exposure was defined using the measurement from a single temperature station in Tsukuba City; however, individual‐level temperature exposure may vary depending on the exact location of residence, as well as quality of housing, occupation and lifestyle, resulting in potential temperature exposure misclassification. Sixthly, subsequent heat‐related illness could be missed in outpatient settings because only the first date of diagnosis would be recorded for the same conditions treated at the same institution in Japan. Finally, we did not have enough statistical power to look at whether the interaction between antihypertensives and extreme temperature provokes rarer outcomes, such as renal or cardiovascular outcomes, which may result from antihypertensive‐induced dehydration [30, 31].

### 4.5 Policy Implications

Our findings indicate that people taking antihypertensives are more vulnerable to heat‐related illnesses during heatwaves. Monitoring and risk mitigation strategies should be implemented for this patient group during heatwaves. Most cases of heat‐related illnesses are preventable through simple measures, such as avoiding peak heat, staying adequately hydrated and ensuring rooms are kept cool and ventilated [32]. For those who develop symptoms, rapid cooling (preferably cold water immersion) and oral or intravenous fluids with electrolytes and sugars are recommended [33].

## Author Contributions

M.H., A.Y.S.W., M.I., E.W., C.T.R., R.M. and N.T. conceived the study. M.H., A.Y.S.W., M.I., E.W., C.T.R., R.M. and N.T. designed the methodology. M.I., C.K. and Y.T. acquired the data and assisted with exposure, covariate and outcome definitions. M.H. undertook the analyses, with support from A.Y.S.W., M.I., C.K., Y.T. and N.T. All contributing authors assisted with interpretation of the results. M.H. prepared the first draft of the manuscript. All contributing authors reviewed and edited the manuscript.

## Funding

This work was supported by the Medical Research Council (MRC) (MR/N013638/1), Barts Charity (MGU0504) and Wellcome Trust (225868/Z/22/Z). Funders had no role in the study design, collection, analysis and interpretation of data; in the writing of the report and in the decision to submit the article for publication.

## Ethics Statement

This study was approved by the Ethics Committee of the University of Tsukuba (approval numbers: 1666‐1). The consent to access data was waived because the study is an anonymised secondary‐data study.

## Consent

The analyses used only de‐identified patient‐level data, and therefore individual informed consent was not required.

## Conflicts of Interest

I.J.D. has received research grants from GlaxoSmithKline (GSK) and AstraZeneca and holds shares in GSK. The other authors declare no conflicts of interest.

## Supporting information


**Table S1:** International Classification of Diseases 10th Revision‐10 (ICD‐10) clinical codes.
**Table S2:** Heat‐related illness clinical codes.
**Table S3:** Antihypertensives identified in Japanese claims data.
**Table S4:** Start and end dates of heatwaves identified in Tsukuba City between 2014 and 2019.
**Table S5:** Median daily temperature (IQR) by month in Tsukuba City 2014–2019.
**Table S6:** Average daily temperature during heatwave periods by year (2014–2019).
**Figure S1:** Graph showing daily average, minimum and maximum temperature in Tsukuba City.
**Table S7:** Sensitivity analysis: risk of heat‐related illness at different temperatures with and without antihypertensives excluding colder months (November–March).
**Table S8:** Sensitivity analysis: risk of heat‐related illness at different temperatures with and without antihypertensives excluding hospitalisation periods.
**Table S9:** Sensitivity analysis: risk of heat‐related illness in the total SCCS study population regardless of medication use, stratified by age and sex.

## Data Availability

The data that support the findings of this study are available from the University of Tsukuba. Restrictions apply to the availability of these data, which were used under license for this study. Data are available from the author(s) with the permission of University of Tsukuba.
