# Supplementary material for: Antihypertensive Medications and Risk of Heat‐Related Illness During Heatwaves: Self‐Controlled Case Series
Source: Pharmacoepidemiol Drug Saf. 2026 Sep 8;35(9):e70447. doi: 10.1002/pds.70447 (PMC13553137; doi:10.1002/pds.70447)
Supplement: Supplementary file 1 [file PDS-35-e70447-s001.docx]

**Supplementary tables and figures**

[Table S1. International Classification of Diseases 10th Revision-10 (ICD-10) clinical codes	2](#_Toc227603533)

[Table S2. Heat-related illness clinical codes	3](#_Toc227603534)

[Table S3. Antihypertensives identified in Japanese claims data	4](#_Toc227603535)

[Table S4. Start and end dates of heatwaves identified in Tsukuba City between 2014-2019	5](#_Toc227603536)

[Table S5. Median daily temperature (IQR) by month in Tsukuba City 2014-2019	7](#_Toc227603537)

[Table S6. Average daily temperature during heatwave periods by year (2014-2019)	8](#_Toc227603538)

[Figure S1. Graph showing daily average, minimum and maximum temperature in Tsukuba City	9](#_Toc227603539)

[Table S7. Sensitivity analysis: risk of heat-related illness at different temperatures with and without antihypertensives excluding colder months (November-March)	13](#_Toc227603540)

[Table S8. Sensitivity analysis: risk of heat-related illness at different temperatures with and without antihypertensives excluding hospitalisation periods	14](#_Toc227603541)

[Table S9. Sensitivity analysis: risk of heat-related illness in the total SCCS study population regardless of medication use, stratified by age and sex	15](#_Toc227603542)

# Table S1. International Classification of Diseases 10th Revision-10 (ICD-10) clinical codes

| **ICD-10 code** | **Term** |
| --- | --- |
| I10 | Essential (primary) hypertension |
| I11 | Hypertensive heart disease |
| I11.0 | Hypertensive heart disease with (congestive) heart failure |
| I11.9 | Hypertensive heart disease without (congestive) heart failure |
| I12 | Hypertensive renal disease |
| I12.0 | Hypertensive renal disease with renal failure |
| I12.9 | Hypertensive renal disease without renal failure |
| I13 | Hypertensive heart and renal disease |
| I13.0 | Hypertensive heart and renal disease with (congestive) heart failure |
| I13.1 | Hypertensive heart and renal disease with renal failure |
| I13.2 | Hypertensive heart and renal disease with both (congestive) heart failure and renal failure |
| I13.9 | Hypertensive heart and renal disease, unspecified |
| I15 | Secondary hypertension |
| I15.0 | Renovascular hypertension |
| I15.1 | Hypertension secondary to other renal disorders |
| I15.2 | Hypertension secondary to endocrine disorders |
| I15.8 | Other secondary hypertension |
| I15.9 | Secondary hypertension, unspecified |
| I67.4 | Hypertensive encephalopathy |

# Table S2. Heat-related illness clinical codes

| **ICD-10 code** | **Term** |
| --- | --- |
| T67 | Heatstroke and sunstroke |
| E86 | Volume depletion |
| E87 | Disorders of fluid, electrolyte and acid-base balance |
| X30 | Exposure to excessive natural heat |

# Table S3. Antihypertensives identified in Japanese claims data

| **Drug class** | **WHO ATC** | **Drug name** |
| --- | --- | --- |
| Diuretics (C03) | C03DA01 | spironolactone |
|  | C03AA06 | trichlormethiazide |
|  | C03DA04 | eplerenone |
|  | C03XA01 | tolvaptan |
|  | C03BA11 | indapamide |
|  | C03DA02 | potassium canrenoate |
|  | C03BA05 | mefruside |
|  | C03AA03 | hydrochlorothiazide |
|  | C03BA09 | meticrane |
| Calcium channel blocker (C08) | C08CA01 | amlodipine |
|  | C08CA05 | nifedipine |
|  | C08CA14 | cilnidipine |
|  | C08CA15 | benidipine |
|  | C08DB01 | diltiazem |
|  | C08DA01 | verapamil |
|  | C08CA04 | nicardipine |
|  | C08CA10 | nilvadipine |
|  | C08CA07 | nisoldipine |
|  | C08CA08 | nitrendipine |
|  | C08CA11 | manidipine |
|  | C08EA02 | bepridil |
|  | C08CA12 | barnidipine |
|  | C08CA02 | felodipine |
| ACE inhibitors/ ARBs (C09) | C09CA06 | candesartan |
|  | C09CA07 | telmisartan |
|  | C09CA03 | valsartan |
|  | C09CA08 | olmesartan medoxomil |
|  | C09CA09 | azilsartan medoxomil |
|  | C09CA01 | losartan |
|  | C09CA04 | irbesartan |
|  | C09AA02 | enalapril |
|  | C09AA03 | lisinopril |
|  | C09AA16 | imidapril |
|  | C09AA14 | temocapril |
|  | C09AA10 | trandolapril |
|  | C09AA04 | perindopril |
|  | C09AA06 | quinapril |
|  | C09XA02 | aliskiren |
|  | C09AA08 | cilazapril |
|  | C09AA01 | captopril |
|  | C09AA07 | benazepril |
|  | C09AA12 | delapril |

# Table S4. Start and end dates of heatwaves identified in Tsukuba City between 2014-2019

| **Heatwave start** | **Heatwave end** | **Duration, days** |
| --- | --- | --- |
| 23/07/2014 | 27/07/2014 | 4 |
| 31/07/2014 | 07/08/2014 | 7 |
| 19/08/2014 | 22/08/2014 | 3 |
| 13/07/2015 | 15/07/2015 | 2 |
| 19/07/2015 | 22/07/2015 | 3 |
| 25/07/2015 | 27/07/2015 | 2 |
| 31/07/2015 | 07/08/2015 | 7 |
| 18/07/2016 | 19/07/2016 | 1 |
| 04/08/2016 | 10/08/2016 | 6 |
| 25/08/2016 | 26/08/2016 | 1 |
| 05/09/2016 | 06/09/2016 | 1 |
| 11/07/2017 | 17/07/2017 | 6 |
| 20/07/2017 | 22/07/2017 | 2 |
| 06/08/2017 | 09/08/2017 | 3 |
| 23/08/2017 | 25/08/2017 | 2 |
| 15/07/2018 | 23/07/2018 | 8 |
| 01/08/2018 | 02/08/2018 | 1 |
| 22/08/2018 | 26/08/2018 | 4 |

# Table S5. Median daily temperature (IQR) by month in Tsukuba City 2014-2019

|  | **2014** | **2015** | **2016** | **2017** | **2018** | **2019** | **2014-2019** |
| --- | --- | --- | --- | --- | --- | --- | --- |
| **January** | 2.1 (1.1-4.4) | 3.7 (2.2 - 5.2) | 4.2 (2.6 - 4.8) | 3.0 (1.4 - 4.8) | 2.1 (0.7 - 3.9) | 2.8 (2.0 - 4.1) | 3.1 (1.6 - 4.7) |
| **February** | 2.7 (1.8 - 5.2) | 3.4 (2.6 - 5.3) | 4.3 (2.9 - 5.8) | 4.5 (3.7 - 6.5) | 3.3 (1.8 - 5.0) | 5.3 (3.5 - 7.1) | 3.9 (2.6 - 5.7) |
| **March** | 7.7 (4.6 - 10.9) | 8.0 (6.6 - 10.7) | 8.0 (5.5 - 11.2) | 6.9 (5.3 - 7.7) | 10.4 (7.3 - 12.5) | 8.1 (6.9 - 10.8) | 7.8 (6.2 - 10.8) |
| **April** | 13.0 (10.5 - 15.1) | 13.6 (10.1 - 15.7) | 14.3 (12.6 - 15.1) | 13.8 (11.6 - 15.3) | 15.8 (13.2 - 17.3) | 13.3 (8.4 - 15.3) | 13.8 (11.5 - 15.7) |
| **May** | 18.7 (16.1 - 19.8) | 19.5 (18.3 - 20.7) | 18.4 (17.1 - 20.3) | 18.5 (16.8 - 20.7) | 19.2 (16.9 - 20.6) | 18.6 (17.6 - 19.6) | 18.8 (17.1 - 20.3) |
| **June** | 21.9 (20.6 - 23.0) | 21.3 (20.1 - 22.2) | 21.4 (19.8 - 22.8) | 21.3 (19.2 - 22.3) | 20.9 (19.6 - 23.1) | 21.1 (19.4 - 22.5) | 21.4 (20.0 - 22.7) |
| **July** | 25.3 (22.9 - 27.1) | 26.9 (21.5 - 27.9) | 24.8 (22.7 - 26.2) | 27.2 (25.1 - 27.8) | 27.8 (26.7 - 29.4) | 23.0 (20.7 - 26.4) | 26.1 (23.0 - 27.6) |
| **August** | 26.3 (23.0 - 28.5) | 26.1 (23.1 - 27.8) | 26.4 (25.4 - 27.3) | 24.8 (23.4 - 27.6) | 27.6 (24.8 - 28.9) | 28.2 (25.4 - 29.1) | 26.4 (24.7 - 28.3) |
| **September** | 20.7 (19.7 - 22.1) | 21.4 (20.1 - 23.1) | 23.6 (22.4 - 25.5) | 21.6 (20.8 - 22.7) | 21.9 (20.4 - 23.6) | 23.9 (21.2 - 25.9) | 22.1 (20.5 - 23.9) |
| **October** | 16.4 (14.3 - 18.8) | 16.5 (15.7 - 17.8) | 16.4 (14.5 - 19.3) | 14.7 (12.8 - 19.0) | 16.6 (15.4 - 20.2) | 17.7 (16.5 - 20.0) | 16.6 (14.8 - 19.1) |
| **November** | 11.3 (8.9 - 13.0) | 12.8 (10.7 - 14.1) | 9.9 (8.0 - 11.9) | 9.3 (7.8 - 11.4) | 11.8 (9.9 - 14.4) | 11.7 (10.1 - 13.0) | 11.1 (8.9 - 13.0) |
| **December** | 3.9 (2.2 - 5.5) | 6.8 (4.9 - 9.2) | 6.5 (4.1 - 8.7) | 3.7 (2.8 - 5.4) | 5.4 (2.8 - 9.0) | 6.4 (4.7 - 8.2) | 5.3 (3.3 - 7.7) |

# Table S6. Average daily temperature during heatwave periods by year (2014-2019)

|  | 2014 | 2015 | 2016 | 2017 | 2018 | 2019 | 2014-2019 |
| --- | --- | --- | --- | --- | --- | --- | --- |
| **Pre-heatwave** |  |  |  |  |  |  |  |
| Min, max | 18.9, 35.1 | 17.4, 34.8 | 17.8, 32.4 | 17.7, 34.6 | 14.3, 33.9 | 23.1, 34.2 | 14.3, 35.1 |
| Median (IQR) | 25.3 (22.6 - 26.9) | 26.9 (26.3 - 28.1) | 25.9 (25.2 - 26.1) | 25.7 (24.4 - 27.3) | 27.1 (23.8 - 28.0) | 27.8 (27.3 - 28.6) | 26.2 (24.8 - 27.5) |
| **Heatwave** |  |  |  |  |  |  |  |
| Min, max | 21.8, 36.2 | 22.1, 36.1 | 21.8, 36.0 | 21.6, 35.0 | 23.5, 37.4 | 23.7, 35.5 | 21.6, 37.4 |
| Median (IQR) | 28.6 (28.0 - 28.9) | 28.7 (28.3 - 29.2) | 27.3 (27.1 - 27.8) | 28.2 (27.6 - 28.3) | 29.6 (29.4 - 30.0) | 29.2 (28.8 - 29.6) | 28.6 (28.0 - 29.4) |
| **Post-heatwave** |  |  |  |  |  |  |  |
| Min, max | 18.8, 34.6 | 22.0, 36.1 | 16.3, 31.1 | 19.0, 34.6 | 20.0, 35.4 | 22.0, 33.3 | 16.3, 36.1 |
| Median (IQR) | 25.8 (23.8 - 27.0) | 27.4 (26.8 - 28.1) | 24.8 (22.6 - 25.4) | 25.1 (23.6 - 27.4) | 26.7 (24.8 - 27.7) | 26.9 (25.4 - 28.2) | 26.1 (24.7 - 27.5) |

# Figure S1. Graph showing daily average, minimum and maximum temperature in Tsukuba City

**Table 2. Main SCCS analysis: risk of heat-related illness at different temperatures with and without antihypertensives**

|  | **With antihypertensive** | | | | **Without antihypertensive** | | | | **P-value** |
| --- | --- | --- | --- | --- | --- | --- | --- | --- | --- |
|  | **Events** | **PYs** | **IRR** | **95% CIs** | **Events** | **PYs** | **IRR** | **95% CIs** |  |
| Baseline | 11603 | 28486.38 | 1 | - | 4371 | 9625.73 | 1 | - | <0.01 |
| Pre-heatwave | 692 | 1137.38 | 1.28 | 1.18-1.39 | 231 | 391.74 | 1.10 | 0.96-1.26 |  |
| Heatwave | 1187 | 1501.75 | 1.64 | 1.53-1.75 | 386 | 500.4 | 1.42 | 1.27-1.58 |  |
| Post-heatwave | 962 | 1435.67 | 1.41 | 1.31-1.52 | 271 | 489.98 | 1.03 | 0.91-1.17 |  |

**Table 5. Subgroup SCCS analyses: Risk of heat-related illness at different temperatures with and without individual antihypertensive classes**

A. **ACE inhibitors/ ARBs**

|  | **With ACE inhibitors/ARBs** | | | | **Without ACE inhibitors/ARBs** | | | | **P-value** |
| --- | --- | --- | --- | --- | --- | --- | --- | --- | --- |
|  | **Events** | **PYs** | **IRR** | **95% CIs** | **Events** | **PYs** | **IRR** | **95% CIs** |  |
| Baseline | 7097 | 18071.41 | 1 | - | 8749 | 20015.16 | 1 | - | <0.01 |
| Pre-heatwave | 410 | 719.64 | 1.26 | 1.14-1.40 | 513 | 810.74 | 1.26 | 1.14-1.38 |  |
| Heatwave | 770 | 954.78 | 1.77 | 1.63-1.92 | 803 | 1047.39 | 1.50 | 1.39-1.62 |  |
| Post-heatwave | 644 | 910.34 | 1.57 | 1.44-1.71 | 598 | 1017.8 | 1.17 | 1.07-1.28 |  |

B. **Calcium channel blockers**

|  | **With CCBs** | | | | **Without CCBs** | | | | **P-value** |
| --- | --- | --- | --- | --- | --- | --- | --- | --- | --- |
|  | **Events** | **PYs** | **IRR** | **95% CIs** | **Events** | **PYs** | **IRR** | **95% CIs** |  |
| Baseline | 8938 | 22053.03 | 1 | - | 6984 | 16042.43 | 1 | - | <0.01 |
| Pre-heatwave | 548 | 877.29 | 1.33 | 1.21-1.46 | 373 | 652.08 | 1.13 | 1.01-1.26 |  |
| Heatwave | 920 | 1156.51 | 1.68 | 1.56-1.81 | 650 | 845.63 | 1.49 | 1.37-1.62 |  |
| Post-heatwave | 721 | 1105.18 | 1.40 | 1.29-1.51 | 509 | 820.87 | 1.22 | 1.11-1.34 |  |

C. **Diuretics**

|  | **With diuretics** | | | | **Without diuretics** | | | | **P-value** |
| --- | --- | --- | --- | --- | --- | --- | --- | --- | --- |
|  | **Events** | **PYs** | **IRR** | **95% CIs** | **Events** | **PYs** | **IRR** | **95% CIs** |  |
| Baseline | 2153 | 4398.57 | 1 | - | 13674 | 33679.52 | 1 | - | 0.38 |
| Pre-heatwave | 106 | 174.41 | 1.08 | 0.89-1.32 | 814 | 1357.45 | 1.27 | 1.18-1.38 |  |
| Heatwave | 211 | 230.59 | 1.59 | 1.37-1.84 | 1363 | 1771.59 | 1.62 | 1.52-1.72 |  |
| Post-heatwave | 157 | 219.88 | 1.27 | 1.07-1.49 | 1093 | 1710.47 | 1.36 | 1.27-1.46 |  |

# Table S7. Sensitivity analysis: risk of heat-related illness at different temperatures with and without antihypertensives excluding colder months (November-March)

|  | **With drug** | | | | **Without drug** | | | | **P-value** |
| --- | --- | --- | --- | --- | --- | --- | --- | --- | --- |
|  | **Events** | **PYs** | **IRR** | **95% CIs** | **Events** | **PYs** | **IRR** | **95% CIs** |  |
| Baseline | 5918 | 13839. | 1 | - | 1944 | 4226.11 | 1 | - | <0.01 |
| Pre-heatwave | 716 | 1170.17 | 1.26 | 1.17-1.37 | 208 | 358.98 | 1.11 | 0.96-1.28 |  |
| Heatwave | 1232 | 1543.38 | 1.64 | 1.53-1.75 | 341 | 458.78 | 1.40 | 1.24-1.57 |  |
| Post-heatwave | 989 | 1476.58 | 1.39 | 1.30-1.50 | 243 | 448.88 | 1.03 | 0.90-1.18 |  |

# Table S8. Sensitivity analysis: risk of heat-related illness at different temperatures with and without antihypertensives excluding hospitalisation periods

|  | **With drug** | | | | **Without drug** | | | | **P-value** |
| --- | --- | --- | --- | --- | --- | --- | --- | --- | --- |
|  | **Events** | **PYs** | **IRR** | **95% CIs** | **Events** | **PYs** | **IRR** | **95% CIs** |  |
| Baseline | 12555 | 29394.06 | 1 | - | 4009 | 8936.47 | 1 | - | 0.02 |
| Pre-heatwave | 599 | 1137.97 | 1.00 | 0.92-1.09 | 161 | 330.38 | 0.89 | 0.76-1.05 |  |
| Heatwave | 1066 | 1501.49 | 1.33 | 1.25-1.43 | 287 | 422.45 | 1.23 | 1.09-1.40 |  |
| Post-heatwave | 856 | 1434.99 | 1.14 | 1.06-1.23 | 206 | 413.83 | 0.92 | 0.80-1.06 |  |

# Table S9. Sensitivity analysis: risk of heat-related illness in the total SCCS study population regardless of medication use, stratified by age and sex

|  | **Events** | **PYs** | **IRR** | **95% CIs** |
| --- | --- | --- | --- | --- |
| Baseline | 15707 | 38061.45 | 1 | - |
| Pre-heatwave | 930 | 1533.1 | 1.24 | 1.16-1.34 |
| Heatwave | 1573 | 2002.18 | 1.59 | 1.50-1.69 |
| Post-heatwave | 1252 | 1932.25 | 1.33 | 1.25-1.42 |

| **Sex** | **Females** | | **Males** | |  |
| --- | --- | --- | --- | --- | --- |
|  | **IRR** | **95% CIs** | **IRR** | **95% CIs** | **P-interaction** |
| Baseline | 1 | - | 1 | - | 0.89 |
| Pre-heatwave | 1.22 | 1.11-1.34 | 1.28 | 1.15-1.42 |  |
| Heatwave | 1.58 | 1.46-1.70 | 1.62 | 1.49-1.76 |  |
| Post-heatwave | 1.33 | 1.22-1.44 | 1.34 | 1.22-1.47 |  |

| **Age group, years** | **18-39** | | **40-49** | | **50-59** | | **60-69** | | **≥70** | |  |
| --- | --- | --- | --- | --- | --- | --- | --- | --- | --- | --- | --- |
|  | **IRR** | **95% CIs** | **IRR** | **95% CIs** | **IRR** | **95% CIs** | **IRR** | **95% CIs** | **IRR** | **95% CIs** | **P-interaction** |
| Baseline | 1 | - | 1 | - | 1 | - | 1 | - | 1 | - | 0.98 |
| Pre-heatwave | 1.37 | 0.72-2.62 | 1.66 | 1.05-2.63 | 1.46 | 1.06-2.01 | 1.25 | 1.07-1.47 | 1.25 | 1.07-1.47 |  |
| Heatwave | 1.40 | 0.79-2.48 | 1.64 | 1.09-2.49 | 1.69 | 1.29-2.21 | 1.66 | 1.46-1.88 | 1.66 | 1.46-1.88 |  |
| Post-heatwave | 1.20 | 0.65-2.23 | 1.35 | 0.85-2.13 | 1.21 | 0.88-1.66 | 1.34 | 1.17-1.54 | 1.34 | 1.17-1.54 |  |
